# Incidence and Mortality Rates and Clinical Characteristics of Type 1 Diabetes among Children and Young Adults in Cochabamba, Bolivia

**DOI:** 10.1155/2017/8454757

**Published:** 2017-08-29

**Authors:** Elizabeth Duarte Gómez, Gabriel Andrew Gregory, Miriam Castrati Nostas, Angela Christine Middlehurst, Alicia Josephine Jenkins, Graham David Ogle

**Affiliations:** ^1^Centro Vivir con Diabetes, Av. Simón López, No. 375, Cochabamba, Bolivia; ^2^International Diabetes Federation Life for a Child Program, Glebe, NSW 2037, Australia; ^3^NHMRC Clinical Trials Centre, University of Sydney, Sydney, NSW 2006, Australia; ^4^Diabetes NSW, Glebe, NSW 2037, Australia

## Abstract

**Objectives:**

To determine incidence, mortality, and clinical status of youth with diabetes at the Centro Vivir con Diabetes, Cochabamba, Bolivia, with support from International Diabetes Federation Life for a Child Program.

**Methods:**

Incidence/mortality data analysis of all cases (<25 year (y)) diagnosed January 2005–February 2017 and cross-sectional data (December 2015).

**Results:**

Over 12.2 years, 144 cases with type 1 diabetes (T1D) were diagnosed; 43.1% were male. Diagnosis age was 0.3–22.2 y; peak was 11-12 y. 11.1% were <5 y; 29.2%, 5–<10 y; 43.1%, 10–<15 y; 13.2%, 15–<20 y; and 3.5%, 20–<25 y. The youngest is being investigated for monogenic diabetes. Measured incidence in Cercado Province (Cochabamba Department) was 2.2/100,000 children < 15 y/y, with ≈80% ascertainment, giving total incidence of 2.7/100,000 children < 15 y/y. Two had died. Crude mortality rate was 2.3/1000 patient years. Clinical data on 141 cases <35 y: mean/median HbA1c was 8.5/8.2% (69/62 mmol/mol), levels higher in adolescents. Three were on renal replacement therapy; four others had substantial renal impairment. Elevated BMI, triglycerides, and cholesterol were common: 19.1%, 18.3%, and 39.1%, respectively.

**Conclusions:**

Bolivia has low T1D incidence. Reasonable glycemic control is being achieved despite limited resources; however, some have serious complications and adverse cardiovascular risk factor profiles. Further attention is needed for complications.

## 1. Introduction

For any nation, understanding the epidemiology and clinical status of young people with diabetes is essential for training health professionals and also for conducting advocacy with the respective government to plan and improve the appropriate clinical services.

Very little published material is available on diabetes in youth in Bolivia. The International Diabetes Federation (IDF) Atlas estimates of children < 15 years with type 1 diabetes in Bolivia [1] are extrapolated from the data from Peru that is over two decades old [2, 3], a time during which the incidence of type 1 diabetes has been increasing at 2-3% or more per annum in many countries [1].

In 2005, the IDF Life for a Child (LFAC) Program [4], with the assistance of Rotary International, commenced support for the Centro Vivir con Diabetes (CVcD), a multidisciplinary clinic in Cochabamba. Satellite networks were established in five other cities. LFAC Program commenced professional mentoring and provision of insulin (initially with support of Insulin for Life Australia [5]). Later, blood glucose meters and test strips (three tests/day), HbA1c testing, and educational materials were provided.

This study presents epidemiological data on all young people with diabetes known to CVcD from January 2005 to February 2017 and the clinical features of the cohort receiving care at the end of 2015 and documents the initial impact of the LFAC Program.

## 2. Materials and Methods

The study was approved by the Board of Centro Vivir con Diabetes.

### 2.1. Epidemiology Data

Data (age, gender, and diabetes type) were compiled on all people diagnosed with diabetes at age < 25 years (y) and seen by CVcD and its five satellite clinics from January 2005 to February 2017. Diabetes was diagnosed according to the standard World Health Organization (WHO) criteria [6], and the diabetes type was determined by the local investigators based on clinical features. It is not possible to determine the exact national incidence as other diabetes care providers exist in Bolivia and no secondary ascertainment sources are available. However, in Cochabamba Province—comprising Cochabamba city and its immediate environs—where 63 (43.8%) of cases were living at diagnosis, CVcD is well-known and the ascertainment rate is estimated at 80%. A minimum incidence for Cercado Province was calculated according to census data [7], with population interpolated or extrapolated according to the annual growth rate of 1.6% [8].

The crude mortality rate was calculated as the total number of deaths in the cohort of 144 subjects divided by the total number of years from diagnosis until the last follow-up (February 2017) or, if they had died, date of death. Results are expressed as mortality per 1000 patient years.

### 2.2. Clinical Status

Clinical information was collated on all 141 subjects being followed from September to December 2015, who aged <25 y at diabetes diagnosis and ≤35 y at the time of this assessment. These 141 subjects included some cases diagnosed before January 2005 and excluded some cases included in the incidence study who were now being seen by other centres. The data were collected via LFAC Annual Clinical Data Sheets. Data included sex, date of birth, diabetes diagnosis date, details of diabetes treatment regimen, and physical and biochemistry measures. Also, social parameters as to whether diabetes was limiting school attendance, if subjects were in the age-appropriate grade, and how well overall the young person was, in their doctor's opinion, psychologically coping with their diabetes (rated as good, with some problems, or poor) were also recorded. Body weight and height were measured by electronic scales and a stadiometer, respectively, with subjects wearing light-weight clothing and without shoes. Body mass index (BMI) was then calculated. BMI SD scores were calculated using the WHO standards for <5 years [9], >5 years, and <19 years [10]. Blood pressure SD score was calculated from published data from the United States [11].

The presence of cataracts, retinopathy, and peripheral neuropathy was recorded. However, the methodology was not prescribed and therefore could have varied across centres and physicians.

### 2.3. Biochemistry

Glycosylated haemoglobin (HbA1c), serum creatinine, total cholesterol, triglycerides, and HDL cholesterol were measured in local biochemistry laboratories, with the exception of HbA1c at Cochabamba where it was measured with a Siemens DCA Vantage machine (Erlangen, Germany). Lipid tests were done on fasting samples. LDL cholesterol was calculated using the Friedewald equation and hence is only available on the subset of patients who had HDL cholesterol levels [12].

Estimated glomerular filtration rate (eGFR) was calculated for subjects ≥ 14 y by the CKD-EPI (Chronic Kidney Disease Epidemiology Collaboration) formula [13], adjusted for individual surface area.

Initial impact of Life for a Child Program was measured by assessing all HbA1c levels of all subjects having data at baseline (program commencement in 2004) and then at either six- or 12-month follow-up.

### 2.4. Statistics

Data and descriptive statistics were managed in Excel and analysed in R 3.3.1 (R Core Team, Vienna, Austria) with RStudio integrated development environment (RStudio Team, Boston, USA).

## 3. Results

### 3.1. Epidemiological Data

#### 3.1.1. Sex Distribution and Age of Onset

**Figure 1:**
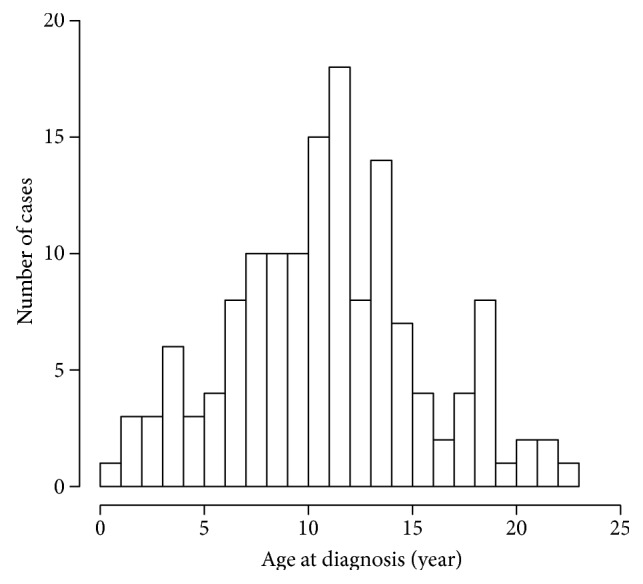
Age at diagnosis of type 1 diabetes in Bolivia, 2005–2017.

Of the 144 subjects diagnosed at age < 25 y between January 2005 and February 2017, 62 (43.1%) were males. Age of onset ranged from 0.3–22.2 y, with a peak at 11 years (see Figure 1 histogram). Sixteen subjects (11.1%) were diagnosed at age 0–4 y, 42 (29.2%) were diagnosed at age 5–9 y, 62 (43.1%) were diagnosed at age 10–14 y, 19 (13.2%) were diagnosed at age 15–19 y, and 5 (3.5%) were diagnosed at age 20–24 y.

Genetic studies are being arranged for the child diagnosed at 3 months to check for a monogenic form of diabetes. This child has no other medical problems.

#### 3.1.2. Minimum Incidence

Sixty-three cases of diabetes (diagnosed at <25 years of age) occurred in the Cercado Province of Cochabamba Department in the 12.2 y study period from January 2005 to February 2017. Forty-nine were <15 y at diagnosis and 14 were 15–24 y. This is a measured incidence of 2.2 per 100,000 children < 15 y/y. Ascertainment is estimated as 80%, resulting in an estimated total incidence of 2.7 per 100,000 children < 15 y/y.

#### 3.1.3. Mortality Rate

Of the 144 subjects followed up for 0.1–12.2 y (mean ± SD 5.9 ± 3.4 y), two had died, a male aged 22.8 y from gastric ulcer haemorrhage and a female aged 13.4 y from ketoacidosis (who had been abandoned by her family). The crude mortality rate was 2.3/1000 patient years.

### 3.2. Clinical Status

#### 3.2.1. Demographics

One hundred and forty-one T1D subjects diagnosed at <25 years of age and ≤35 y were being followed up by CvCD at the end of 2015. Sixty-four (45%) patients were male. Sixty (43%) were from Cochabamba Department; 31 (22%), Santa Cruz; 27 (19%), La Paz; 12 (8.5%), Sucre; 6 (4.3%), Tarija; and 5 (3.5%), Potosi. One child also had cerebral palsy, one had Down syndrome, and one child also had epilepsy. Age at diagnosis and diabetes duration are shown in Table 1.

#### 3.2.2. Diabetes Care

Ninety-four subjects (67%) were adjusting their insulin dose (data available on 141 subjects), with the remainder giving a fixed insulin dose set in agreement with their treating clinicians. Regarding insulin types (*n* = 138), 99 (72%) were on short- and long-acting human insulin only. Twenty-five (18%) were using analogue insulin, of which nine (7%) were using analogue only. Thirteen (9%) were on premixed insulin, and one patient was receiving long-acting human insulin only.

For the number of insulin injections per day (*n* = 122), 18 subjects (158%) were taking two injections per day; 70 (57%), three injections; 24 (20%), four injections; and the remaining 10 (8%), five injections. Insulin usage ranged 0.35–1.76 units/kg/day (mean ± SD 0.99 ± 0.29) (*n* = 136).

The frequency of self-blood glucose tests per week (*n* = 139) was 14–28 (mean ± SD 21.3 ± 1.4). The frequency of clinic visits in the last year (*n* = 131) was 1–8 (mean ± SD 3.5 ± 1.3).

#### 3.2.3. Social Parameters

School/college attendance was regular in 93 (96%) of the 97 patients for whom this information was available, and the year of schooling was age-appropriate in 94%. Out of 105 patients for whom a response was recorded on the question of how well they thought their patients were coping with their diabetes, 62 (59%) of the responses were “good,” 38 (37%) were with “some problems,” and four (4%) were “poor.”

#### 3.2.4. Physical Measurements

Height and weight information was available for 137 patients, and 81 of these were under 19 years, permitting BMI SD score calculation (shown in Table 1).

Blood pressure was measured on 21 subjects < 20 years, with systolic and diastolic SD scores shown in Table 1. Both systolic and diastolic mean SD scores were above zero (*p* < 0.001 for both, by one-sided *t*-test). None of the patients for whom SD scores were available were noted to have hypertension (as defined by local clinical standards) or be on antihypertensive medications.

#### 3.2.5. HbA1c

HbA1c was measured in the 135 subjects with duration of diabetes > 6 months. Mean and median HbA1c levels were 8.5% (69 mmol/mol) and 8.2% (66 mmol/mol), respectively (see also Table 1). Forty-four subjects (33%) had HbA1c levels in the recommended target range of <7.5% (58 mmol/mol); 35 (25%), 7.5%–8.49% (58–69 mmol/mol); 48 (36%), 8.5%–12% (69–108 mmol/mol); and the remaining eight subjects (5.8%), >12% (108 mmol/mol). Twenty-six percent of those aged <15 y and 30% of those aged 15–24 y had target HbA1c < 7.5% (58 mmol/mol). A series of one-way ANOVAs on log-transformed HbA1c levels were performed, finding trends towards relationships between HbA1c levels, the number of insulin injections per day (*p* = 0.07), and the use of analogue insulin (*p* = 0.06), but not for gender (*p* = 0.38).

**Figure 2:**
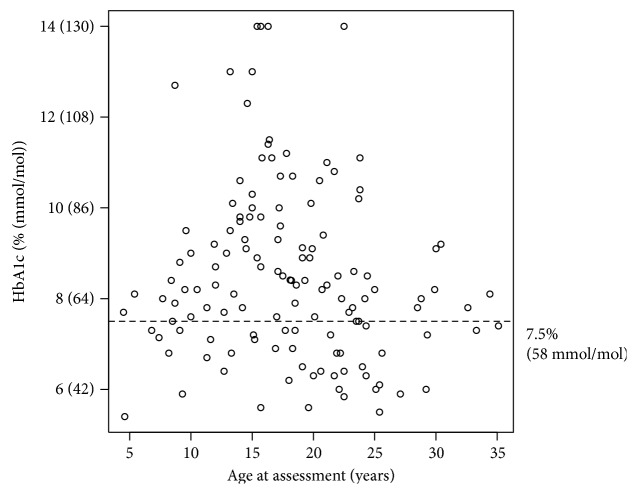
Relationship of age to HbA1c.

A pattern of increased HbA1c levels and variance during adolescence was observed from a plot of HbA1c by age (Figure 2). For those aged 0–13 y, the HbA1c mean ± SD was 7.9% (63 mmol/mol) ± 1.3% (14 mmol/mol); for those aged 13–20 y, the HbA1c mean ± SD was 9.4% (78 mmol/mol) ± 2.0% and for those aged >20 y, the HbA1c mean ± SD was 7.9% (63 mmol/mol) ± 1.7% (18 mmol/mol). This indicates a significant difference in HbA1c levels between age groups (*p* = 0.0002 by one-way ANOVA on log-transformed values). However, Levene's test for heterogeneity of variance was not statistically significant (*p* = 0.24).

Mean/median HbA1c levels by a diabetes clinic site were 9.0%/8.4% (75/68 mmol/mol) in Cochabamba, 7.9%/7.6% (63/60 mmol/mol) in La Paz, 8.0%/7.4% (64/57 mmol/mol) in Potosi, 8.5%/8.4% (69/68 mmol/mol) in Santa Cruz, 8.5%/8.3% (69/67 mmol/mol) in Sucre, and 7.1%/6.8% (54/51 mmol/mol) in Tarija, with no statistical difference between clinics on ANOVA.

#### 3.2.6. Lipids

Lipid levels are shown in Table 1. No subjects were on lipid-lowering agents. For total cholesterol, 97 patients (80%) had <200 mg/dL (5.2 mmol/L), 16 patients (13%) had 200–239 mg/dL (5.2–6.2 mmol/L), and nine (7.3%) had ≥240 mg/dL (≥6.2 mmol/L). Total cholesterol levels were not correlated with concurrent HbA1c levels (*p* = 0.14 by Pearson's correlation).

Triglyceride levels (*n* = 121) were <1.7 mmol/L (150 mg/dL) in 99 patients (82%), 1.7–2.2 mmol/L (150–199 mg/dL) in 11 (9%) patients, and 2.3–5.6 mmol/L (200–499 mg/dL) in 11 (9%) patients (all subjects had <4.5 mmol/L (450 mg/dL). Log-transformed triglyceride levels were correlated with concurrent HbA1c levels (*p* = 0.03 by Pearson's correlation).

HDL cholesterol was measured in 27 patients from the Department of La Paz (see Table 1). None of these patients were on renal replacement therapy. Fifteen patients (56%) had HDL < 1.0 mmol/L (40 mg/dL) for men or 1.3 mmol/L (50 mg/dL) for women. There were no significant correlations between HDL cholesterol and either HbA1c or log-transformed triglyceride levels.

Seven patients (26%) had calculated LDL cholesterol levels < 100 mg/dL (<2.6 mmol/L), 10 patients (37%) had 100–129 mg/dL (2.6–3.3 mmol/L), and 10 patients (37%) had ≥130 mg/dL (3.3 mmol/L).

Table 2 shows the percentage of subjects meeting target levels for cardiovascular risk factors (BMI, triglycerides, total cholesterol, and HbA1c), for the 115 subjects that had values for all four measurements.

#### 3.2.7. Renal Function

Serum creatinine values are shown in Table 1. Five patients (4%) had serum creatinine levels above the recommended level of 1.3 mg/dL (115 *μ*mol/L) for males or 1.1 mg/dL (97 *μ*mol/L) for females. eGFR for the 115 subjects ≥ 14 y ranged from 10.4–178.9 mL/min/1.73m^2^ (mean ± SD 104.9 ± 31.0 mL/min/1.73m^2^). Three patients on renal replacement therapy (see below) were excluded from this analysis.

Two subjects were receiving haemodialysis (age 28.5 and 28.8 y) (duration of diabetes to start of haemodialysis 16.5 and 1.25 y), one had received a renal transplant after 0.3 y on haemodialysis (age 30.4 y, duration of diabetes to time of haemodialysis 17.3 y) and the other was noted as having chronic renal failure (24.4 y, duration 20.5 y, eGFR 12.8 mL/min/1.73m^2^). Three other subjects had eGFR < 50 mL/min/1.73m^2^, all with diabetes duration of at least 16 years. Two of these three were receiving treatment for hypertension, and one was also treated with erythropoietin injections for associated anaemia.

Seventeen subjects ≥ 14 y had an eGFR of ≥140 mL/min/1.73m^2^, suggestive of hyperfiltration.

#### 3.2.8. Other Complications

One subject had hypothyroidism which was being treated. One subject had experienced a severe hypoglycemia episode in the past resulting in severe cognitive and motor impairment. Three patients were recorded as having diabetic retinopathy, and five subjects had cataracts (two of whom also had retinopathy). Neuropathy (loss of vibration and light touch) was recorded in one subject.

### 3.3. Initial Impact of Life for a Child Program

**Figure 3:**
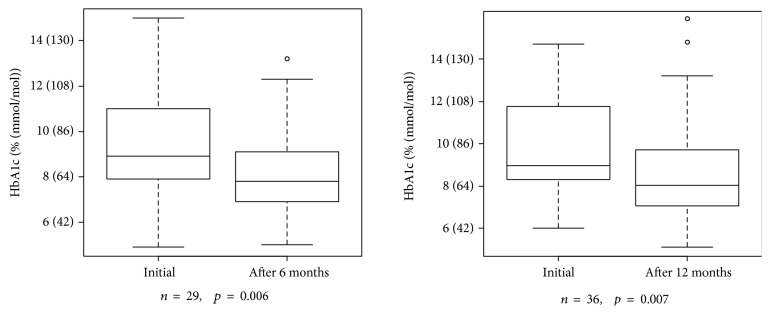
Initial impact at commencement of Life for a Child support.

HbA1c results at the start of the LFAC Program in 2004-2005 were available for 57 subjects. Values ranged from 4.9–15% (30–140 mmol/mol), with mean ± SD 9.6 ± 2.2% (81 ± 24 mmol/mol). For six-month paired data, mean ± SD HbA1c at baseline was 9.4 ± 2.3% (79 ± 25 mmol/mol), and mean ± SD HbA1c at six months was 8.1 ± 1.8% (65 ± 20 mmol/mol) (*n* = 29, *p* = 0.006 by one-sided paired *t*-test). For 12-month paired data, mean ± SD HbA1c at baseline was 9.7 ± 2.1% (83 ± 23 mmol/mol), and mean ± SD HbA1c at twelve months was 8.7 ± 2.5% (72 ± 27 mmol/mol) (*n* = 36, *p* = 0.007 by one-sided paired *t*-test) (see Figure 3).

## 4. Discussion

Globally, the incidence rates of type 1 diabetes in children vary widely due to differences in genetic susceptibility, combined with inadequately understood but powerful and evolving environmental factors [1–3].

There have been no previous studies on type 1 diabetes incidence in Bolivia. Rates vary substantially in Central/South American and other Hispanic populations. Estimates for Bolivia in the IDF Atlas [1] are extrapolated from a 1990–1991 study in Lima, Peru, which found an incidence of 0.5 per 100,000 children < 15 y/y [2]. Other studies in South America in the early 1990s (all <15 y) showed rates per 100,000 ranging from 0.1 in Caracas, Venezuela, to 8.3 in Montevideo [2, 3]. Countries with both recent and older data are seeing sharp rises in type 1 diabetes incidence—in Mexico, from 3.4 (per 100,000) in 2000 to 6.2 in 2012 for young people < 20 y in the Instituto Mexicano del Seguro Social (IMSS) health insurance service [14]; in Chile, from 5.7 in 2006 to 12.1 in 2012 for those aged <20 y [15], with the sharpest increase in the <5 y age group; and in Brazil, a trend for increased incidence in those aged <15 y from 1986 to 2006 [16].

Further afield for Hispanic populations, rates (<15 y) are 20.6 in Spain (<15 y, 1995–2001) [1], 13.2 in Portugal (<15 y, 1994–1998) [1], and 14.1 in Hispanic Whites in the USA (<15 y, 2002–2003) [17], with a recent study showing an increase of 4.2% per year in 2003–2012 on numbers < 20 y [18].

Some of the variance in South American populations is thought to be due to lower rates of type 1 diabetes in Central/South American indigenous peoples as compared to those of Hispanic White/mixed descent [2, 19]. Larenas et al. [20] found 3.8 times higher rates in Caucasian Chileans than in Mapuche (native Chileans). Bolivia has, by a substantial margin, the highest proportion of indigenous peoples in Central/South America [21]. This lower rate in indigenous populations is likely largely genetic in origin due to differences in HLA haplotypes [19, 22, 23]. However, differing exposure to environmental factors may also be potent influencers: rates of type 1 diabetes have increased in many countries as standards of living have increased. Incidence rates were found to be higher in high socioeconomic communes in Santiago de Chile [24].

The observed type 1 diabetes rate in Cercado in this study—2.2 per 100,000 children < 15 y/y with an estimated ascertainment of 80%, giving an estimated incidence of 2.7 per 100,000—is five times higher than the figure previously used for Bolivia (based on the Peruvian data from 1990–1991 [2]. It is likely that type 1 incidence is increasing in Bolivia; however, further studies will be needed to confirm this. This is still a low incidence rate compared to global figures. The female preponderance seen in Bolivia is common in low incidence studies [25] and is believed to be due to fathers with type 1 susceptibility genes being more likely to survive and pass on diabetogenic genes than mothers, with fathers' genes more likely to cause type 1 diabetes in female offspring and mothers' genes to cause diabetes in male offspring. In addition, it is thought that fathers' genes may be more diabetogenic than similar genes in mothers [26, 27].

Monogenic diabetes has been reported from various countries, and it is likely that the child diagnosed at 3 months of age has this form of diabetes—genetic testing is being arranged. If there is a gene defect, alternate noninsulin therapy may be possible depending on the specific gene [28].

Like various other less-resourced countries [29], the Bolivian government health service does not cover the cost of care for people with diabetes at any age. Most families cannot afford the cost of care, which can be prohibitive in such countries for families with lower incomes [30], and this can translate into the premature death of the child or young adult with diabetes, as occurred once in this Bolivian cohort. Furthermore, in Bolivia, there are very few paediatric endocrinologists and, due to its relative rarity, limited knowledge of type 1 among general paediatricians. Therefore, the support from a centre such as CvCD ± LFAC is crucial for families with limited resources. CvCD provides a multidisciplinary service of not just health professionals in diabetology but also health professionals in ophthalmology, nutrition, psychology, social work, foot care and podiatry, physiotherapy and rehabilitation, laboratory services, and pharmacy. There is also a young group or people with type 1 diabetes which encourage and support each other through meetings and the use of the social media platform “WhatsApp.” The IDF Life for a Child Program provides insulin and, since 2009, also provides blood glucose test strips, educational materials, and point-of-care HbA1c testing in Cochabamba.

The initial impact seen in the LFAC follow-up data from 2004–2005 has been sustained. Mortality is low at 2.3 per 1000 patient years, and the mean/median HbA1c of 8.5/8.2% (69/62 mmol/mol) is surprisingly good, considering the substantial challenges faced in caring for these young people. Twenty-seven percent of those aged <15 y and 28% of those aged 15–24 y were achieving the recommended HbA1c target of <7.5% (58 mmol/mol). This is not substantially different from the mean of 29/30% (male/female) for subjects aged <15 y and from the mean of 24/20% (male/female) for those aged 15–24 y in a large international study in which 15 of 16 countries were high-income nations [31].

The higher HbA1c values seen in adolescents/emerging adults are similar to those of other reports [32] and likely reflect lifestyle and behavioural factors and growth spurt-related insulin resistance. As in other countries, thoughtful medical attention and support are warranted during this period, along with provision of insulin supply security.

It is of great concern that three young people with diabetes required renal replacement therapy and four others have substantial renal impairment (eGFR <50 mL/1.73m^2^). Furthermore, 17 had hyperfiltration, which may be a risk factor for diabetic nephropathy, although recent evidence suggests this may not be the case [33, 34]. The presence of ESRD at a young age suggests that blood glucose control must have been poor for some years, and we note that for most of the Bolivian subjects evaluated herein, regular self-blood glucose monitoring has only been possible since 2009. Anderzén et al. [35] has shown the importance of a good early start in terms of blood glucose control, if later complications are to be reduced.

Further assessment should be done for early signs of nephropathy such as microalbuminuria and hypertension, with management (with ACE inhibitors or LDL-lowering “statin” drugs) being informed by the forthcoming results of the Adolescent Type 1 Diabetes Cardio-Renal Intervention Trial (ADDIT) [36]. Given the number with renal complications, it is likely that a thorough systematic assessment would lead to more subjects being found with other microvascular complications such as neuropathy and retinopathy, for whom early intervention with risk factor control and increased screening rates is appropriate.

The common cause of death of people with type 1 diabetes is cardiovascular disease, for which dyslipidaemia is a major risk factor and is treatable, with significant LDL-C, cardiovascular event, and mortality reduction with statins [37]. The substantial numbers with one or more lipid risk factors such as high triglycerides and total cholesterol and low HDL cholesterol levels, combined with the suggestion of blood pressure elevation, raise the possibility that some patients have features of the metabolic syndrome, which is recognised in type 1 diabetes and associated with higher rates of diabetes vascular complications [38, 39]. This “double diabetes” may benefit from the addition of metformin to insulin and risk factor control [40], though clinical trials with vascular end-points are still awaited.

The study has three other limitations. It is possible that with further study (autoantibodies and C-peptide, unfortunately not available in this clinical context), some of the subjects diagnosed with type 1 diabetes may be recategorised as having type 2 diabetes. Secondly, HbA1c measurements were not standardised across clinics, and so it is possible that results could be affected by the different methods used. Finally, the 80% ascertainment is an estimated figure, and Cercado Province may not be fully representative of Bolivia—incidence may vary in other parts of the country due to ethnic or socioeconomic factors.

## 5. Conclusion

This study has estimated the incidence of type 1 diabetes in Bolivia, confirming that incidence is relatively low compared to that in many other countries. The results demonstrate that reasonable blood glucose control and low mortality can be achieved in many subjects in a low-resourced setting with international support. However, there is a significant risk of early long-term complications in some subjects, as evidenced by renal impairment and substantial levels of cardiovascular risk factors.

## Figures and Tables

**Table 1:** Clinical and biochemical characteristics.

Parameter	*n*	Mean (SD)	Range
Age at diagnosis (years)	141	9.9 (4.4)	0.3–22.2
Diabetes duration (years)	141	8.1 (5.7)	0.1–26.9
BMI SD score	137	0.9 (1.1)	−2.2–3.7
Systolic SD score	21	0.7 (0.8)	−1.4–2.0
Diastolic SD score	21	1.1 (0.7)	−0.1–2.6
HbA1c (%)^∗^	135	8.5 (1.9) %	5.4–14.0%
HbA1c (mmol/mol)^∗^	135	69 (21) mmol/mol	36–130 mmol/mol
Serum creatinine (mg/dL)	130	0.92 (0.69)	0.5–6.2
Total cholesterol (mg/dL)	122	167 (43.2)	81–334
Triglycerides (mg/dL)	121	112 (63)	42–497
HDL cholesterol (mg/dL)	27	43 (14.2)	7.9–59
LDL cholesterol (mg/dL, calculated)	27	124 (38)	43–191
Non-HDL cholesterol (mg/dL, calculated)	27	149 (44)	58–216

^∗^HbA1c not included on six subjects assessed within six months of diagnosis.

**Table 2:** Risk factors for cardiovascular disease.

Risk factor	Definition	*n*	%
High BMI	For <19 years, SD score ≥ 2.0; if ≥19 years, BMI ≥ 25	22	19.1
Elevated fasting triglycerides	≥150 mg/dL (≥1.7 mmol/L)	21	18.3
Elevated fasting total cholesterol	≥175 mg/dL (≥4.5 mmol/L)	45	39.1
HbA1c above target range	≥7.5% (58 mmol/mol)	74	64.3
One risk factor	—	44	38.3
Two risk factors	—	34	29.6
Three risk factors	—	14	12.2
Four risk factors	—	2	1.7

(*n* = 115).
